# Gadoxetic acid-enhanced MRI in primary sclerosing cholangitis: added value in assessing liver function and monitoring disease progression

**DOI:** 10.1007/s00261-020-02731-z

**Published:** 2020-09-12

**Authors:** Aboelyazid Elkilany, Dominik Geisel, Tobias Müller, Andreas Fischer, Timm Denecke

**Affiliations:** 1 Department of Diagnostic and Interventional Radiology, Charité-Universitätsmedizin Berlin, corporate member of Freie Universität Berlin, Humboldt-Universität zu Berlin, and Berlin Institute of Health, Augustenburger Platz 1, 13353 Berlin, Germany; 2 Division of Gastroenterology and Hepatology, Department of Medicine, Charité-Universitätsmedizin Berlin, corporate member of Freie Universität Berlin, Humboldt-Universität zu Berlin, and Berlin Institute of Health, Augustenburger Platz 1, 13353 Berlin, Germany; 3 Department of Diagnostic and Interventional Radiology, Universitätsklinikum Leipzig, Leipzig, Germany

**Keywords:** Primary sclerosing cholangitis, Gadoxetic acid, Contrast enhancement, Functional magnetic resonance imaging

## Abstract

**Purpose:**

To investigate the added value of gadoxetic acid-enhanced MRI in monitoring liver function and disease progression in patients with primary sclerosing cholangitis (PSC).

**Methods:**

We retrospectively identified 104 consecutive patients (75 males; mean age 41.98 ± 12.5 years) with confirmed diagnosis of PSC who underwent 227 gadoxetic acid-enhanced MRI examinations between January 2008 and May 2019. Relative enhancement (RE) of the liver was correlated with the results of liver function tests (LFTs), scoring models (Model for End-Stage Liver Disease (MELD) score, Mayo Risk Score (MRS), Amsterdam-Oxford model (AOM)), and qualitative MRI findings. In addition, results were analyzed separately for excretory MRI examinations (*n* = 164) and nonexcretory examinations (*n* = 63) depending on excretion of gadoxetic acid into the common bile duct in the hepatobiliary phase (HBP).

**Results:**

There was a significant correlation of RE with MRS (*r* = − 0.652), MELD score (*r* = − 0.474), AOM (*r* = − 0.468), and LFTs (*P* < 0.001). RE and albumin were significantly higher in the excretory group whereas scoring models, bilirubin, aspartate aminotransferase, alkaline phosphatase, and international normalized ratio were lower (*P* < 0.001). RE was lower in segments with absent HBP gadoxetic acid excretion into dilated bile ducts, reduced HBP parenchymal enhancement, atrophy, T2 hyperintensity, and bile duct abnormalities (*P* < 0.001).

**Conclusion:**

Relative enhancement of the liver in gadoxetic acid-enhanced MRI can be used to evaluate global and regional liver function and monitor disease progression in patients with PSC. Hepatobiliary phase gadoxetic acid biliary excretion appears to be a reproducible qualitative parameter for evaluating disease severity that can be easily integrated into routine clinical practice.

**Electronic supplementary material:**

The online version of this article (10.1007/s00261-020-02731-z) contains supplementary material, which is available to authorized users.

## Introduction

Primary sclerosing cholangitis (PSC) is a rare, chronic cholestatic liver disease of unknown etiology that is characterized by progressive diffuse inflammation, obliterating fibrosis, stricture formation, and destruction of the intra- and extrahepatic bile ducts and will ultimately progress to liver cirrhosis and end-stage liver disease [1–3].

Different clinical, laboratory, histologic, and cholangiographic scoring models have been developed to estimate disease severity and predict the clinical course of PSC and patient outcome. Other potential applications of these prognostic scoring models include prediction of the response to therapy and risk stratification following therapeutic interventions [4–7]. The Mayo risk score (MRS) was designed specifically for assessing the short-term (4-year) mortality risk of PSC patients. However, the MRS was developed in a group of patients with end-stage liver disease and is not suitable for use in early PSC. In addition, it cannot predict survival of individual patients [4, 5, 8–10]. The Amsterdam cholangiographic classification system is limited clinically by its invasive nature [11, 12]. The Model for End-Stage Liver Disease (MELD) score is a valid prognostic score for prediction of both the short- and intermediate-term mortality risk of patients with chronic liver disease [13–15]. The Amsterdam-Oxford model (AOM), the most recently recommended prognostic model, can predict long-term transplant-free survival in PSC patients [4, 16].

Because of its invasiveness and associated complications, endoscopic retrograde cholangiopancreatography (ERCP) is now restricted to therapeutic interventions in patients with PSC. Magnetic resonance imaging (MRI), including magnetic resonance cholangiopancreatography (MRCP), has replaced ERCP as the imaging modality of choice for diagnosis of PSC [6, 17, 18].

Gadoxetic acid is a hepatocyte-specific contrast agent that allows not only morphological but also functional evaluation of global and regional liver and consequently can be used as a prognostic marker in patients with PSC [5, 19]. Biliary excretion, which represents around 50% of gadoxetic acid excretion in individuals with normal liver and kidney function, is delayed in patients with impaired liver function and biliary obstruction [20, 21].

The purpose of our study is to investigate gadoxetic acid-enhanced MRI as a surrogate imaging-based model for evaluation of liver function and disease progression in patients with PSC, focusing on relative enhancement (RE) of the liver and gadoxetic acid biliary excretion in the hepatobiliary phase (HBP).

## Materials and methods

### Patient population and study design

We retrospectively identified 126 consecutive patients with confirmed diagnosis of PSC who underwent gadoxetic acid-enhanced MRI examinations (*n* = 268) at our institution between January 2008 and May 2019 from the picture archiving and communication system (PACS) and patients’ electronic medical records. The diagnosis of PSC was established in accordance with the guidelines of the European Association for the Study of the Liver (EASL) [17, 22]. This retrospective study was approved by the institutional review board. Informed consent was waived.

Inclusion criteria were a confirmed diagnosis of PSC according to EASL guidelines and completion of the MRI examination (including MRCP). Exclusion criteria were: history of cholangiocarcinoma, liver transplantation (LTx), liver resection or locoregional liver intervention for management of hepatic malignancy, and nondiagnostic image quality due to severe artifacts.

**Fig. 1:**
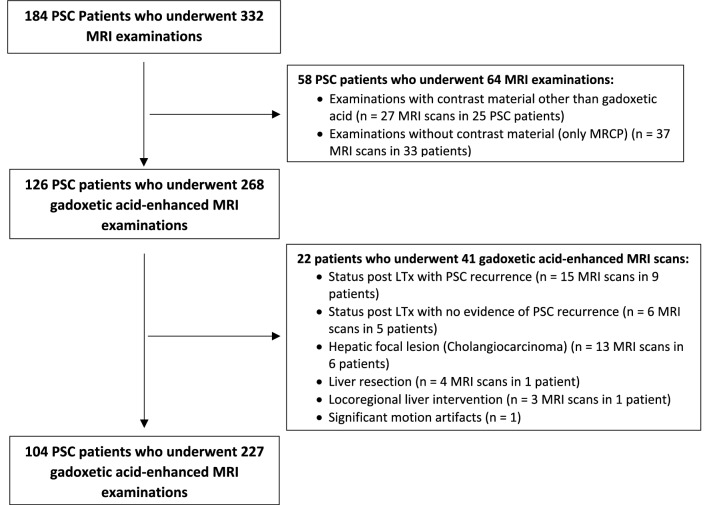
Flow chart of inclusion and exclusion of patients with PSC who underwent gadoxetic acid-enhanced MRI. *LTx* liver transplantation; *MRI* magnetic resonance imaging; *PSC* primary sclerosing cholangitis

After exclusion, 104 patients who underwent 227 MRI examinations remained for analysis (Fig. 1).

### Laboratory parameters and clinical scoring systems

Liver function tests (LFTs) (aspartate aminotransferase [AST], alanine aminotransferase [ALT], alkaline phosphatase [ALP], gamma-glutamyl transferase [GGT], serum total bilirubin, and serum albumin), kidney function tests (serum creatinine and estimated glomerular filtration rate [eGFR]), international normalized ratio (INR), platelets, C-reactive protein (CRP), and white blood cell (WBC) count performed within 1 month before or after gadoxetic acid-enhanced MRI were selected for analysis. In addition, tumor marker (carbohydrate antigen 19-9 [CA 19-9]) was tested in patients with suspected cholangiocarcinoma.

The MELD score (*n* = 207) (based on serum bilirubin, creatinine, and INR) [13], MRS (*n* = 96) (based on patient age, bilirubin, albumin, AST, and history of variceal bleeding) [8], and AOM (*n* = 92) (based on patient age at diagnosis, PSC subtype [large-duct–small-duct], bilirubin, albumin, AST, ALP, and platelet count) [16] including 5-, 10- and 15-year transplant-free estimated survival rates were prospectively calculated using data collected from patients’ electronic medical records.

### MRI examinations

MRI examinations were performed at our institution using 6 different scanners: 1.5T Magnetom Avanto, 1.5T Magnetom Aera, 3.0T Magnetom Skyra, 3.0T Biograph mMR (Siemens Healthcare, Erlangen, Germany), 1.5T Intera (Philips, Best, The Netherlands), and 1.5T GE Signa Excite (GE Medical Systems, Milwaukee, WI, USA). In all examinations, transverse T1-weighted images covering the entire liver with 60–80 slices were acquired before and approximately 20 min after intravenous bolus injection of 0.1 ml/kg body weight of gadoxetic acid (Gd-EOB-DTPA, gadoxetate disodium; Primovist^®^/Eovist^®^, Bayer HealthCare, Berlin, Germany). MRCP and T2-weighted sequences were performed before gadoxetic acid administration. Sequence parameters are listed in Supplementary Table 1.

### Image analysis

All MRI examinations were reviewed by two reader with 8 and 12 years of experience in abdominal imaging and MRI who were blinded to clinical data and laboratory findings.

#### Quantitative analysis

Images were analyzed using a dedicated viewing workstation (Centricity PACS RA1000 version 6.0, General Electric, Milwaukee, WI, USA). Signal intensity (SI) was measured by placing one circular region of interest (ROI) approximately 2.5 cm in diameter in each Couinaud liver segment (8 ROIs). Each ROI was placed in identical locations in images acquired prior to (SI unenhanced) and approximately 20 min after gadoxetic acid administration in the HBP (SI in HBP). Large vessels (caliber > 5 mm), bile ducts, tumor masses, and artifacts were avoided. RE during the HBP was calculated for each segment and the whole liver (i.e., mean SI of the 8 measurements) using the following formula:

$${\text{RE = }}\left( {{\text{SI in HBP {-} SI unenhanced}}} \right){\text{ / SI unenhanced}} .$$

#### Qualitative analysis

Each liver segment was evaluated for the following imaging features: 1-degree of bile duct dilatation, 2-significant bile duct stenosis, 3-bile duct caliber irregularity including beading (multiple segmental caliber irregularities in the form of strictures alternating with dilatations) and pruning (peripheral bile duct attenuations) [23], 4-isolated alterations in parenchymal signal intensity (T2 hyperintensity), 5-egmental lobar atrophy, 6-HBP parenchymal contrast enhancement and 7-HBP gadoxetic acid excretion into dilated segmental bile ducts (Supplementary Table 2).

Relative enhancement was correlated with scoring results (MELD score, MRS, and AOM) and different laboratory values. At the segmental level, RE was correlated with different qualitative imaging findings.

MRI examinations were graded as excretory (164 examinations) versus nonexcretory (63 examinations) depending on HBP biliary excretion of gadoxetic acid into the common bile duct (CBD). These two subgroups were compared regarding RE, results of prognostic scoring models, and laboratory values.

In a subgroup analysis of patients who underwent at least two gadoxetic acid-enhanced MRI examinations, HBP gadoxetic acid biliary excretion into the CBD was evaluated as a predictor of liver function and disease severity based on the changes in RE, scoring results and LFTs (bilirubin, ALP, albumin, INR).

### Statistical analysis

Bivariate analysis (Pearson correlation) was used for simple correlation analysis. The paired *t* test and Mann–Whitney *U*-test were performed to assess differences between two groups. Linear mixed model analysis was used to identify possible predictors of gadoxetic acid biliary excretion during the HBP. Repeated-measures analysis was performed using a linear mixed model. Receiver operating characteristic (ROC) analysis was used to identify cutoff values for different parameters (RE, scoring models, bilirubin, albumin, and ALP) for visualization of HBP gadoxetic acid biliary excretion into the CBD. Statistical analysis was performed with Stata/MP version 16.0 (StataCorp, College Station, Texas, USA). A *P* value < 0.05 was considered statistically significant.

## Results

### Study population

The study included 104 patients (75 males, 29 females; mean age 41.98 ± 12.5 years, age range 15.8–78 years). They had a mean age of 34.4 ± 12.4 years when diagnosed with PSC. Apart from 2 patients (who underwent 3 MRI scans) with small-duct PSC, all patients had large-duct PSC. Patient demographics are presented in Table 1.

**Table 1:** Summary of patient demographics

Variable	*n*	Mean ± SD (min–max)
Female/male	29/75	–
Age at time of MRI acquisition (years)	104	41.98 ± 12.5 (15.8–78)
Age at PSC diagnosis (years)	104	34.4 ± 12.4 (9.5–66)
Amount of contrast medium (mL)	227	7.78 ± 1.19 (5–10)
PSC subtype (large-duct/small-duct)	224/3	–
IBD (UC/Crohn’s disease)	59/9	–
Liver cirrhosis (yes/no)	108/119	–
Variceal bleeding (yes/no)	11/216	–
Bilirubin (mg/dL)	212	1.97 ± 3.04 (0.16–26.25)
AST (U/L)	211	68.67 ± 51.46 (16–405)
ALT (U/L)	213	86.07 ± 84.9 (11–543)
GGT (U/L)	213	234.63 ± 219.06 (9–999)
ALP (U/L)	211	265.12 ± 181.25 (36–1265)
Albumin (gm/L)	100	3.99 ± 0.63 (2.3–5.2)
Platelets (× 10^9^/L)	214	239.04 ± 98.47 (32–593)
INR	209	1.05 ± 0.17 (0.8–2.04)
Creatinine (mg/dL)	215	0.78 ± 0.17 (0.44–1.61)
eGFR (mL/min)	180	92 ± 60.08 (51–888)
CRP (mg/L)	147	12.89 ± 21.56 (0.3–119.2)
Leucocytes(x10^9^/L)	213	6.92 ± 2.74 (2.31–24.65)
CA 19-9 (U/mL)	59	32.79 ± 36.8 (0.2–162)
Mayo risk score	96	0.52 ± 1.43 (− 1.76 to 4.57)
MELD score	207	9 (6–27)
Amsterdam-Oxford model	92	2.05 ± 0.79 (0.80–4.07)
5-year transplant-free estimated survival (%)		83.66 ± 12.92 (35.32–96.13)
10-year transplant-free estimated survival (%)		66.1 ± 21.02 (6.91–90.36)
15-year transplant-free estimated survival (%)		53.43 ± 23.74 (1.18–87.00)
Indications for gadoxetic acid-enhanced MRI		
1. Diagnostic imaging for suspected PSC.	10	–
2. Follow-up of known PSC including evaluation of degree of bile duct stenosis	118	–
3. Screening for suspected focal lesion	93	–
4. Evaluation for LTx	6	–

*ALT* alanine aminotransferase; *AST* aspartate aminotransferase; *ALP* alkaline phosphatase; *CA 19*-*9* carbohydrate antigen 19-9; *CRP* C-reactive protein; *eGFR* estimated glomerular filtration rate; *GGT* gamma-glutamyl transferase; *HBP* hepatobiliary phase; *IBD* inflammatory bowel disease; *INR* international normalized ratio; *MELD* Model for End-Stage Liver Disease; *MRI* magnetic resonance imaging; *PSC* primary sclerosing cholangitis; *UC*, ulcerative colitis

### Analysis of SI and RE of the liver

Mean SI of the liver was 214.32 ± 6.7 before administration of gadoxetic acid (SI unenhanced) and 342.07 ± 12.4 in the HBP. Mean RE was 0.57 ± 0.02. On a lobar level, RE was significantly higher in the right lobe (0.59 ± 0.02) than in the left lobe (0.55 ± 0.02, *P* < 0.001). Patients with PSC complicated by liver cirrhosis (108/227) had significantly lower RE than patients without cirrhosis (0.51 ± 0.02 vs. 0.62 ± 0.02, *P* < 0.001). Analysis of RE in the HBP in relation to the amount of gadoxetic acid administered revealed a significant positive correlation (*r* = 0.156, *P* = 0.019). Supplementary Table 3 provides descriptive results of SI and RE analysis.

### Clinical scores and laboratory findings

With an AOM of 2.05 ± 0.79, the study population had a considerable risk for LTx or death, while a MRS of 0.52 ± 1.43 and a MELD score of 9 indicated an intermediate risk.

**Fig. 2:**
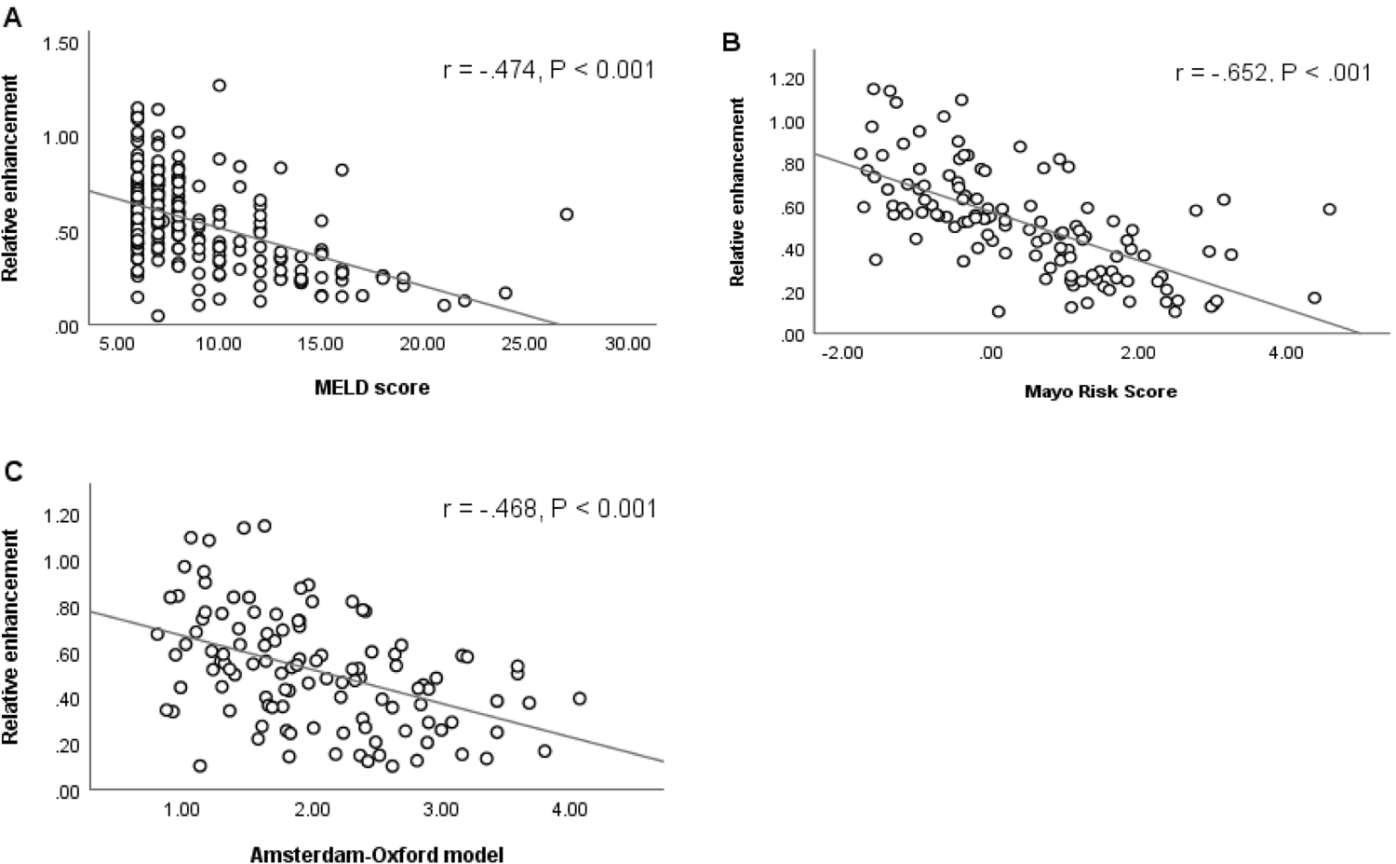
Pearson correlation of mean relative enhancement (RE) of the liver in the hepatobiliary phase with clinical scores. Scatterplot with regression line demonstrates a significant negative correlation (*P* < 0.001) of RE with **a** Model for End-Stage Liver Disease (MELD) score, **b** Mayo risk score, and **c** Amsterdam-Oxford model

Correlation analysis between RE of the whole liver and different clinical scores revealed a significant negative correlation (*P* < 0.001) with MRS (*r* = − 0.652), MELD score (*r* = − 0.474), and AOM (*r* = − 0.468) (Fig. 2) and significant positive correlation (*P* < 0.001) with 5-year, 10-year and 15-year transplant-free survival rates in AOM (Table 2). There was a significant negative correlation between RE and LFTs apart from serum albumin, for which there was a significant positive correlation. (Table 2).

**Table 2:** Summary of Pearson correlation of mean relative enhancement (RE) of the liver in the hepatobiliary phase with model-based scores and laboratory values

	Relative enhancement		
	*n*	Pearson correlation	*P* value
Bilirubin (mg/dL)	212	− 0.404	< 0.001
AST (U/L)	211	− 0.433	< 0.001
ALT (U/L)	213	− 0.227	0.001
ALP (U/L)	211	− 0.409	< 0.001
GGT (U/L)	213	− 0.155	0.024
Albumin (gm/L)	100	0.608	< 0.001
INR	209	− 0.255	< 0.001
Platelets (x10^9^/L)	214	0.068	0.32
Creatinine (mg/dL)	215	0.167	0.01
WBCs (x10^9^/L)	213	− 0.011	0.88
CRP (mg/L)	147	− 0.167	0.04
Mayo risk score	96	− 0.652	< 0.001
MELD score	207	− 0.474	< 0.001
Amsterdam-Oxford model	92	− 0.468	< 0.001
5-year transplant-free estimated survival (%)		0.419	< 0.001
10-year transplant-free estimated survival (%)		0.454	< 0.001
15-year transplant-free estimated survival (%)		0.458	< 0.001

ALT, alanine aminotransferase; AST, aspartate aminotransferase; ALP, alkaline phosphatase; CRP, C-reactive protein; eGFR, estimated glomerular filtration rate; GGT, gamma-glutamyl transferase; INR, international normalized ratio; MELD, Model for End-Stage Liver Disease

*P*-value < 0.05 was considered statistically significant

### Evaluation of gadoxetic acid biliary excretion

**Fig. 3:**
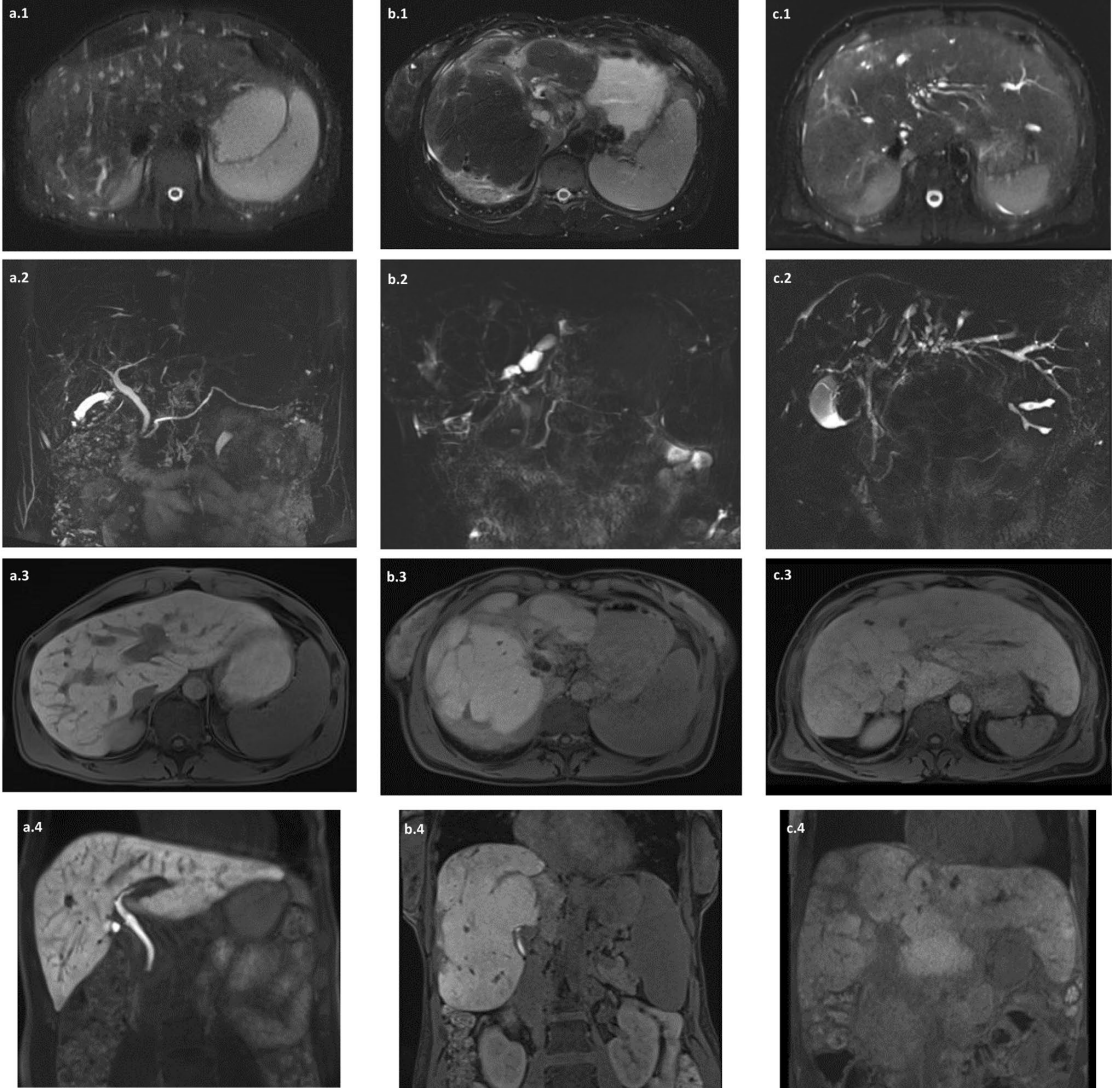
T2-weighted sequence (HASTE) (**a1**, **b1**, **c1**), MRCP (**a2**, **b2**, **c2**), and T1-weighted VIBE sequence 20 min after gadoxetic acid administration (in the HBP) (axial sequence: **a3**, **b3**, **b3** and coronal sequence: **a4**, **b4**, **c4**) in three patients with different stages of PSC. Patient a: Early PSC changes with peripheral bile duct attenuation and slight T2 hyperintensity in the right posterior liver segment. Gadoxetic acid-enhanced images in the HBP demonstrate homogenous parenchymal enhancement and regular excretion of gadoxetic acid in the CBD. Patient b: Typical advanced PSC changes with focal impairment of liver parenchyma in the form of significant segmental bile duct stenosis, T2 hyperintensity, and atrophy affecting most liver segments apart from segments V and VIII. Gadoxetic acid-enhanced images in the HBP demonstrate heterogeneous parenchyma with absent enhancement in affected segments and absent excretion of gadoxetic acid in the CBD. Patient c: End-stage PSC reflected by cirrhotic liver configuration, multisegment bile duct stenosis and atrophy, as well as absent contrast enhancement and excretion of gadoxetic acid in the CBD in the HBP. *CBD* common bile duct; *HASTE* half-Fourier acquisition single-shot turbo spin-echo; *VIBE* volumetric interpolated breath-hold sequence; *HBP* hepatobiliary phase; *PSC* primary sclerosing cholangitis

Regarding the excretion of gadoxetic acid into the CBD in the HBP (Fig. 3), the excretory group had a significantly higher RE and a significantly lower MRS, MELD score, and AOM (*P* < 0.001). Comparison of LFT results between the excretory and nonexcretory group revealed significantly higher levels of serum bilirubin (*P* < 0.001), ALP (*P* < 0.001), AST(*P* = 0.002), and INR (*P* = 0.001) in the nonexcretory group and a significantly higher level of serum albumin (*P* < 0.001) in the excretory group (Table 3).

**Table 3:** Results of comparison between hepatobiliary phase gadoxetic acid biliary excretory and nonexcretory groups

	Nonexcretory group			Excretory group			*P* value
	*n*	Mean	SE	*N*	Mean	SE	
Age at time of MRI (years)	63	47.52	1.61	164	43.2	0.96	0.02
Amount of gadoxetic acid administrated (ml)	63	7.6	0.15	164	7.96	0.09	0.04
Relative liver enhancement (RE)	63	0.38	0.25	164	0.64	0.17	< 0.001
MELD score	61	11	0.65	146	8	0.19	< 0.001
Mayo risk score	34	1.67	0.22	62	− 0.10	0.14	< 0.001
Amsterdam-Oxford model	32	2.52	0.13	60	1.80	0.09	< 0.001
5-year transplant-free estimated survival (%)	32	76.51	2.56	60	87.47	1.33	< 0.001
10-year transplant-free estimated survival (%)	32	53.64	3.87	60	72.74	2.24	< 0.001
15-year transplant-free estimated survival (%)	32	39.51	4.30	60	60.85	2.57	< 0.001
Bilirubin (mg/dL)	61	3.85	0.62	151	1.21	0.1	< 0.001
AST (U/L)	59	89.69	8.65	152	60.51	3.39	0.002
ALT (U/L)	61	96.07	13.72	152	74.18	6.02	0.27
ALP (U/L)	59	342.51	22.13	152	235.09	14.36	< 0.001
GGT (U/L)	61	253.49	28.15	152	227.06	17.76	0.43
Albumin (gm/L)	36	3.51	0.11	64	4.25	0.06	< 0.001
INR	61	1.12	0.03	148	1.01	0.01	0.001
Platelets (x10^9^/L)	62	236.06	15.11	152	240.26	7.24	0.80
Creatinine (mg/dL)	62	0.75	0.20	153	0.80	0.14	0.05

*RE* relative enhancement; *MELD* Model for End-Stage Liver Disease; *AST* aspartate aminotransferase; *ALT* alanine aminotransferase; *ALP* alkaline phosphatase; *INR* international normalized ratio; *GGT* Gamma-glutamyl transferase; *MRI* magnetic resonance imaging; *SE* standard error

*P*-value < 0.05 was considered statistically significant

**Fig. 4:**
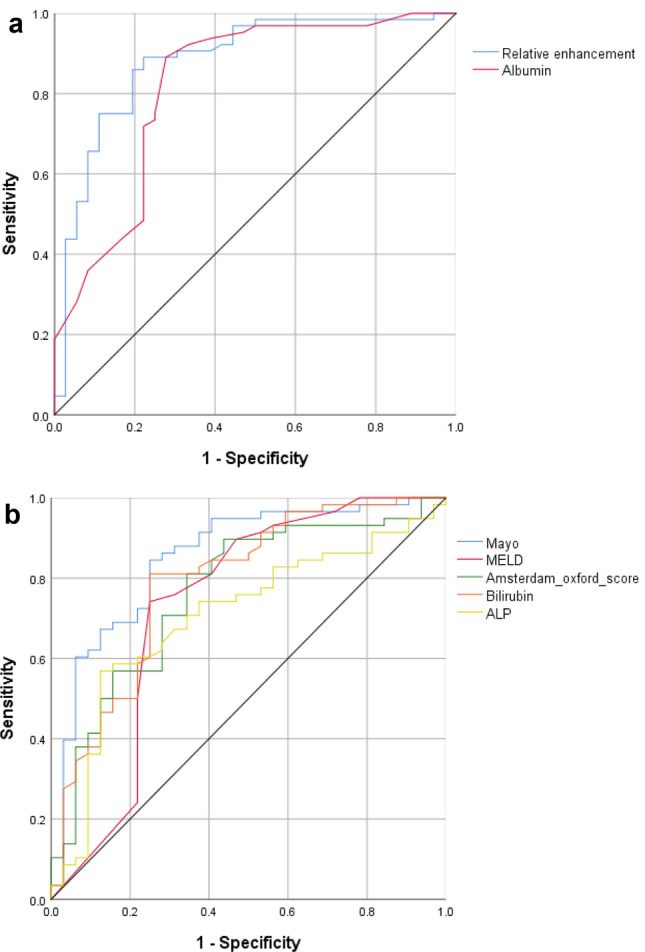
Receiver operating characteristic (ROC) analysis to identify cutoffs for different parameters (relative enhancement, scoring models, bilirubin, albumin, ALP) for visualization of hepatobiliary phase gadoxetic acid biliary excretion into the common bile duct. *ALP* alkaline phosphatase

ROC analysis was performed to identify the parameter most useful for predicting HBP gadoxetic acid biliary excretion into the CBD. Statistical significance was noted for RE, scoring models, and LFTs (bilirubin, albumin, ALP). Relative enhancement had the largest AUC of 0.880 (*P* < 0.001). A cutoff value of 0.43 had 85.9% sensitivity and 80.6% specificity for the presence of HBP gadoxetic acid excretion (Fig. 4, Table 4).

**Table 4:** ROC analysis of relative enhancement (RE), model-based scores, and liver function laboratory values for gadoxetic acid excretion in the hepatobiliary phase

	Cutoff	Sensitivity (%)	Specificity (%)	AUC	SE	*P* value	95% CI	
RE	0.43	85.9	80.6	0.880	0.038	< 0.001	0.806	0.955
Mayo risk score	0.97	84.5	75.0	0.852	0.044	< 0.001	0.766	0.938
MELD score	9.0	74.1	75.0	0.733	0.064	< 0.001	0.608	0.858
Amsterdam-Oxford model	2.37	81.0	65.6	0.765	0.053	< 0.001	0.661	0.870
Bilirubin (mg/dL)	1.86	81.0	75.0	0.789	0.052	< 0.001	0.687	0.0890
Albumin (gm/L)	3.85	89.1	72.2	0.821	0.047	< 0.001	0.728	0.914
ALP (U/L)	262	74.1	62.5	0.703	0.058	0.001	0.590	0.817

*MELD* model for end-stage liver disease score; *ALP* alkaline phosphatase; *SE* standard error; *CI* confidence interval

At the segmental level, analysis of HBP gadoxetic acid excretion into dilated segmental bile ducts revealed significantly higher RE in segments demonstrating contrast excretion (0.68 ± 0.29) than in segments without excretion (0.56 ± 0.31, *P* < 0.001) (Table 5).

**Table 5:** Pairwise comparison of relative enhancement with presence of different features identified by qualitative gadoxetic acid-enhanced MRI evaluation

	Relative enhancement			*P* value in pairwise comparison		
	No	Partial/Subsegmental	Yes (Total/Segmental)	No–Yes (Total)	No–Partial	Partial–Total
Segmental atrophy	0.59 ± 0.31	0.53 ± 0.31	0.49 ± 0.33	0.001	0.73	0.29
Contrast enhancement in HBP	0.47 ± 0.29	0.58 ± 0.32	0.59 ± 0.31	< 0.001	< 0.001	0.61
Segmental T2 hyperintensity	0.58 ± 0.33	0.55 ± 0.32	0.50 ± 0.32	< 0.001	**0.**62	< 0.001
Bile duct caliber irregularity	0.64 ± 0.32	–	0.52 ± 0.33	< 0.001	–	–
Bile duct significant stenosis	0.61 ± 0.33	–	0.50 ± 0.31	< 0.001	–	–
HBP gadoxetic acid excretion into dilated bile ducts	0.56 ± 0.31	–	0.68 ± 0.29	< 0.001	–	–

*HBP* hepatobiliary phase

*P*-value < 0.05 was considered statistically significant

### Analysis of qualitative imaging findings

Analysis of various qualitative MRI findings by liver segment showed RE in the HBP to be significantly higher in segments without bile duct dilatation (0.61 ± 0.34) than in segments with minimal (0.52 ± 0.31, *P* < 0.001), mild (0.42 ± 0.29, *P* < 0.001), or marked bile duct dilatation (0.35 ± 0.29, *P* < 0.001). However, when comparing RE between segments with mild and marked bile duct dilatation, we noted no significant difference (*P* = 0.13). Similarly, RE was significantly lower in segments demonstrating bile duct caliber irregularity, significant bile duct stenosis, atrophy, reduced HBP parenchymal enhancement, or T2 hyperintensity (*P* < 0.001) than in segments without those findings (Table 5).

The results of correlation of RE with qualitative imaging findings at the segmental level are presented in Table 6. The highest correlation was observed with segmental bile duct dilatation (6/8 segments) and HBP gadoxetic acid excretion into dilated segmental bile ducts (5/8 segments), while the lowest correlation was noted with segmental atrophy (1/8 segments).

**Table 6:** Pearson correlation between relative enhancement (RE) in hepatobiliary phase (HBP) and qualitative MRI features at segmental level

RE		Bile ducts				Liver parenchyma		
		Dilatation	Caliber irregularity	Significant stenosis	HBP Contrast excretion into dilated bile ducts	T2 hyperintensity	HBP contrast enhancement	Segmental atrophy
Segment 1	*r*	− 0.127	− 0.169	− 0.111	0.173	− 0.046	0.095	**
	*P* value	0.055	0.011	0.095	0.167	0.488	0.152	**
	*n*	227	227	227	65	227	227	227
Segment 2	*r*	− 0.180	0.005	− 0.083	0.359	− 0.021	0.049	− 0.013
	*P* value	0.008	0.937	0.228	< 0.001	0.755	0.474	0.851
	*n*	214	214	214	124	214	214	214
Segment 3	*r*	− 0.223	− 0.123	− 0.123	0.373	− 0.137	0.152	− 0.102
	*P* value	0.001	0.078	0.079	< 0.001	0.050	0.030	0.144
	*n*	211	211	211	103	211	211	211
Segment 4	*r*	− 0.172	− 0.198	− 0.140	0.179	0.031	0.056	− 0.023
	*P* value	0.010	0.003	0.035	0.091	0.642	0.402	0.732
	*n*	227	227	227	90	227	227	227
Segment 5	*r*	− 0.135	− 0.112	− 0.068	0.165	− 0.086	− 0.021	− 0.087
	*P* value	0.043	0.094	0.308	0.158	0.198	0.749	0.191
	*n*	227	227	227	75	227	227	227
Segment 6	*r*	− 0.276	− 0.120	− 0.177	0.384	− 0.210	0.144	− 0.195
	*P* value	< 0.001	0.072	0.008	0.001	0.001	0.030	0.003
	*n*	227	227	227	78	227	227	227
Segment 7	*r*	− 0.230	− 0.138	− 0.180	0.282	− 0.125	0.126	− 0.105
	*P* value	< 0.001	0.038	0.006	0.003	0.061	0.059	0.115
	*n*	227	227	227	108	227	227	227
Segment 8	*r*	− 0.095	− 0.163	0.047	0.307	− 0.223	0.163	− 0.116
	*P* value	0.155	0.014	0.482	0.006	0.001	0.014	0.081
	*n*	227	227	227	78	227	227	227

*P*-value < 0.05 was considered statistically significant

*r*, Pearson coefficient

**Cannot be computed because at least one of the variables is constant (no segmental atrophy was noted in segment 1)

### Patients with at least two gadoxetic acid-enhanced MRI examinations

Subgroup linear mixed model analysis of patients who underwent at least two gadoxetic acid-enhanced MRI examinations revealed that HBP gadoxetic acid biliary excretion into the CBD was a significant predictor of liver function and disease severity based on the prediction of changes in RE, scoring results (MELD score, MRS, and AOM), and LFTs (bilirubin, albumin, INR) (*P* < 0.001) (Table 7).

**Table 7:** Results of linear mixed model analysis evaluating the effect of hepatobiliary phase (HBP) gadoxetic acid excretion into the common bile duct (CBD) on the temporal changes of different independent variables including relative enhancement (RE), Model for End-Stage Liver Disease (MELD) score, Mayo risk score, Amsterdam-Oxford-PSC score, and laboratory values

Independent variable	Gadoxetic acid excretion into the CBD					
	*B*	SE	*t*-value	*P* value	(95% CI)	
RE of the liver	0.217	0.032	6.59	< 0.001	0.149	0.276
Meld score	− 3.259	0.497	− 6.61	< 0.001	− 4.237	− 2.299
Mayo risk score	− 1.274	0.185	− 6.82	< 0.001	− 1.639	− 0.914
Amsterdam-Oxford model	− 0.431	0.108	− 4.07	< 0.001	− 0.641	− 0.228
10-year transplant-free estimated survival	10.843	2.587	4.22	< 0.001	5.770	15.923
Bilirubin	− 2.463	0.431	− 5.77	< 0.001	− 3.292	− 1.626
Albumin	0.589	0.108	5.69	< 0.001	0.394	0.791
INR	− 0.113	0.029	− 4.37	< 0.001	− 0.160	− 0.062
ALP	− 15.927	23.081	− 0.73	0.42	− 61.151	29.312

*P*-value < 0.05 was considered statistically significant

*ALP* alkaline phosphatase; *INR* international normalized ratio; *B* beta coefficients; *CI* confidence interval; *SE* standard error

**Fig. 5:**
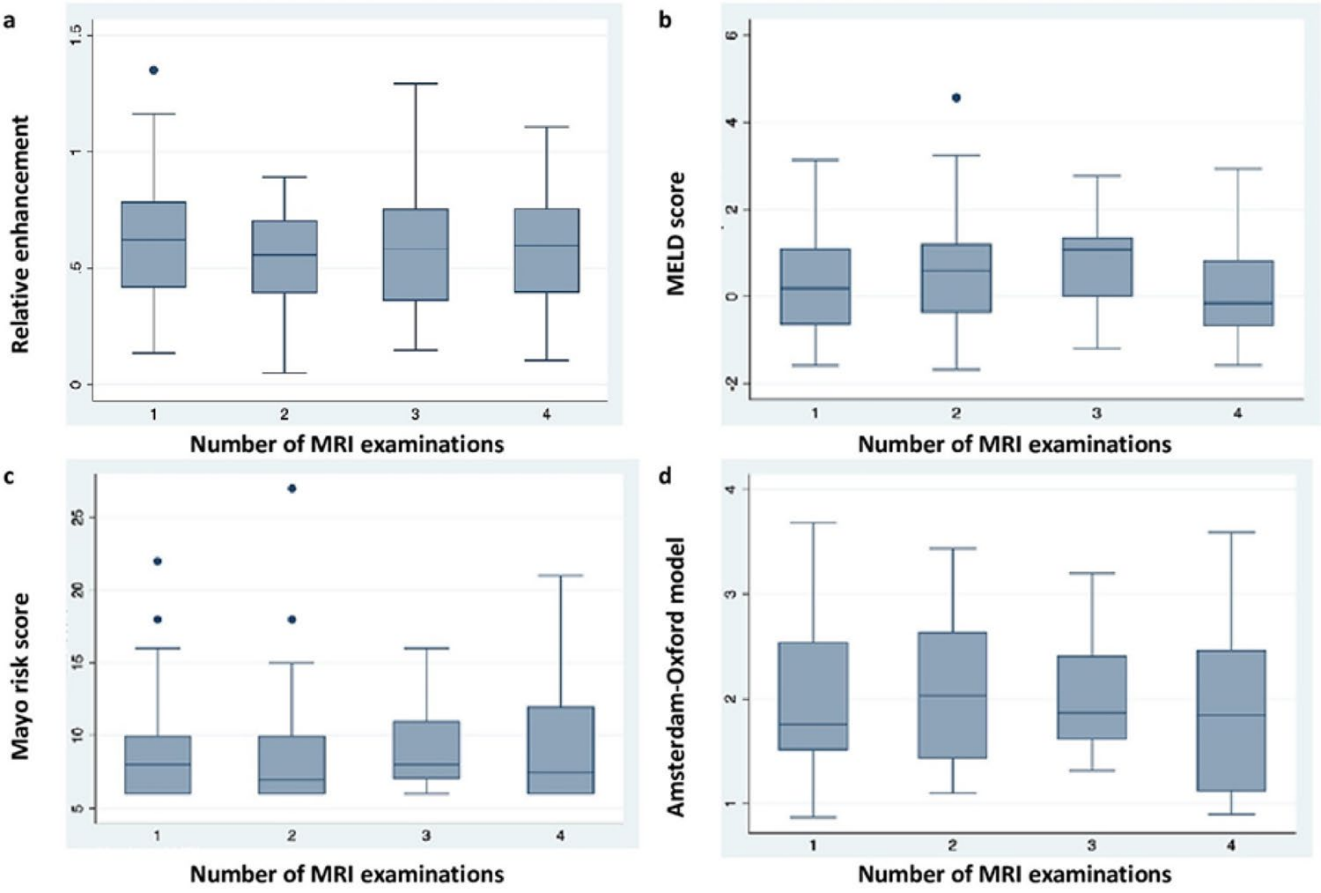
Boxplot diagram demonstrating distribution of **a** relative enhancement, **b** Model for End-Stage Liver Disease (MELD) score, **c** Mayo risk score, and **d** Amsterdam-Oxford model in consecutive gadoxetic acid-enhanced MRI examinations. *MRI* magnetic resonance imaging

In addition, repeated-measures analysis using linear mixed model comparison revealed that RE tended to gradually decrease with increasing scores and bilirubin levels over a longer period of time (Fig. 5). Statistically significant difference in RE was noted between first and second MRI examinations (*P* = 0.03) and this significance was higher in the excretory group (*P* = 0.001) (Supplementary Table 4). Results of descriptive analysis of patients with at least two gadoxetic acid-enhanced MRI examinations are listed in Supplementary Table 5.

## Discussion

Since MRI/MRCP emerged as the modality of choice in the diagnosis and follow-up of patients with PSC, there has been an ongoing interest in developing an MRI-based surrogate parameter for the noninvasive evaluation of disease progression in patients with PSC [24].

The present study investigated gadoxetic acid-enhanced MRI-derived quantitative and qualitative parameters—specifically RE of the liver and gadoxetic acid biliary excretion during the HBP, respectively—as imaging-based surrogate parameters for liver function evaluation (global and regional) and disease progression in patients with PSC.

Relative enhancement of the liver in the HBP correlated significantly with the MRS (moderate correlation), MELD score, and the AOM (low correlation). In addition, RE correlated significantly with LFTs including the suggested surrogate endpoints (bilirubin, ALP, albumin, and INR) [25]. This significant correlation with the scoring models and surrogate endpoints supports the potential of RE as a surrogate prognostic parameter for evaluation of global liver function as well as prediction of short-, intermediate-, and long-term survival.

These findings are consistent with previous studies investigating liver function evaluation in patients with PSC using gadoxetic acid-enhanced MRI-derived indices. Schulze et al. demonstrated a moderate correlation of RE with prognostic scoring models (MELD, MRS, AOM) and LFTs (ALP, albumin, bilirubin, INR). They proposed a RE cutoff of 0.65 for prediction of clinical endpoints with 73.86% sensitivity and 92.86% specificity [26].

Nilsson et al. demonstrated a significant correlation of the hepatic extraction fraction (HEF), input relative blood-flow (irBF), and mean transit time (MTT) with the MRS [5]. Contrary to our results, there was no significant correlation with the MELD score. The most probable explanation might be that they only analyzed a small sample including 12 PSC patients who had predominantly mild disease. Hinrichs et al. investigated T1-mapping in gadoxetic acid-enhanced MRI for evaluating global and regional liver function. They demonstrated a significant correlation of a shorter T1 relaxation time with the MRS, MELD score, AST, bilirubin, and cholinesterase [19].

In the present study, we evaluated the added value of HBP gadoxetic acid biliary excretion into the CBD and/or the duodenum as a qualitative imaging parameter. The group with gadoxetic acid biliary excretion had significantly higher RE, longer estimated transplant-free survival, lower scores in clinical scoring models, and lower levels in LFTs (apart from albumin, which was significantly higher). These findings are in agreement with previous studies performed by Ringe et al. [21] and Nolz et al. [23]. Bastati et al. retrospectively evaluated functional liver imaging score (FLIS) derived from gadoxetic acid-enhanced MRI for estimation of liver function and prediction of transplant-free survival in patients with chronic liver disease. Biliary excretion in the HBP was one of the three FLIS parameters. They found the FLIS to be an independent risk factor for the first hepatic decompensation and mortality [27].

Also, to our knowledge, ours is the first study that analyzed the consistency of liver function over time in PSC patients with at least two gadoxetic acid-enhanced MRI examinations. The analysis revealed HBP gadoxetic acid excretion to be a significant predictor of temporal changes of RE, scoring models, and LFTs (bilirubin, albumin, INR). These findings support the value of HBP gadoxetic acid biliary excretion as a marker of disease severity in patients with PSC.

All of the previously investigated scoring models, surrogate endpoints, and LFTs have only been validated for assessing global liver function, which is not optimal in patients with PSC, which is characterized by heterogeneous distribution of liver function and severity of disease progression. Regional liver function evaluation is paramount for the detection of early PSC, assessment for resectability in patients with cholangiocarcinoma, and for identifying severely affected segments for endoscopic guided drainage or targeting biopsies to reduce sampling errors especially in patients with suspected small-duct PSC [19, 26, 28].

Several findings of the present study provide evidence that segmental liver function in PSC patients can be estimated using RE in gadoxetic acid-enhanced MRI such as the significant difference in RE between right and left hepatic lobes (which could be due to the higher incidence of left lateral segmental atrophy and the effect of gravity which could resulted in a relatively higher enhancement in the right posterior liver segments) and the significantly lower RE in segments with impaired liver parenchyma (no gadoxetic acid excretion within the dilated bile ducts, reduced HBP parenchymal enhancement, atrophy, T2 hyperintensity) and bile duct abnormalities (irregularities, dilatation, significant stenosis).

In addition, these findings support our hypothesis that qualitative findings in gadoxetic acid-enhanced MRI can assist in identifying segments that may benefit from targeted stenting and separate them from segments with already lost function where intervention is not justified. We conclude that the absence of HBP gadoxetic acid excretion into dilated segmental bile ducts combined with the absence of parenchymal enhancement and segmental atrophy indicates segments for which targeted stenting is not advisable and could even be hazardous considering the higher risk of cholangitis associated with retained contrast medium since these segments are not excreting. In contrast, targeted stenting is advised for segments demonstrating gadoxetic acid excretion into the dilated bile ducts, parenchymal enhancement, and no segmental atrophy in the HBP in order to prevent further deterioration of segmental function and thus improve overall liver function capacity. A scoring model including RE, HBP gadoxetic acid biliary excretion, and variable qualitative findings—specifically, bile duct abnormalities—can be used for risk stratification and prediction of disease severity in PSC patients. This approach deserves further investigation in future studies.

*Our study has several limitations* First, we used a retrospective study design. Second, there was no correlation with clinical endpoints such as LTx or death. Third, there might be bias from the use of different MRI scanners and different field strengths. Fourth, we only evaluated gadoxetic acid biliary excretion in the HBP approximately 20 min after gadoxetic acid administration. A second, delayed HBP acquisition at 30–60 min would have added to the validity of our findings considering the well-known fact that HBP excretion of gadoxetic acid is reduced in patients with chronic liver disease. Such a delayed acquisition was previously suggested by Ringe et al. [21] Fifth, we did not correlate the qualitative findings of bile duct abnormalities in T2-weighted MRI/MRCP with ERCP findings or the previously suggested Amsterdam cholangiographic score. We did not consider the latter because it is not widely validated and only takes bile duct abnormalities but not parenchymal changes into account. Sixth, there was no reference standard against which to correlate regional hepatic gadoxetic acid-enhanced MRI measurements. We correlated RE with several qualitative MRI findings. However, the qualitative nature makes them prone to interindividual variation. Finally, in the repeated-measures analysis, and due to the retrospective nature of the study, the exact time point of MRI acquisition was not standardized, which could be another possible cause of bias.

In conclusion, relative enhancement of the liver during the hepatobiliary phase in gadoxetic acid-enhanced MRI can be used to evaluate global and regional liver function and monitor disease progression in patients with PSC. Hepatobiliary phase gadoxetic acid biliary excretion appears to be a reproducible qualitative parameter for evaluating disease severity that can be easily integrated into routine clinical practice.

## Electronic supplementary material

Below is the link to the electronic supplementary material.

Supplementary material 1 (DOCX 48 kb)

## Data Availability

Data and materials used during conducting the study are available. (data transparency).

## Abbreviations

ALT: Alanine aminotransferase
AST: Aspartate aminotransferase
ALP: Alkaline phosphatase
CBD: Common bile duct
ERCP: Endoscopic retrograde cholangiopancreatography
GGT: Gamma-glutamyl transferase
HBP: Hepatobiliary phase
INR: International normalized ratio
LFT: Liver function test
MELD: Model for End-Stage Liver Disease
MRS: Mayo Risk Score
PSC: Primary sclerosing cholangitis
RE: Relative enhancement
SI: Signal intensity
