# Correction to: Gadoxetic acid‑enhanced MRI in primary sclerosing cholangitis: added value in assessing liver function and monitoring disease progression

**DOI:** 10.1007/s00261-021-03155-z

**Published:** 2021-07-05

**Authors:** Aboelyazid Elkilany, Dominik Geisel, Tobias Müller, Andreas Fischer, Timm Denecke

**Affiliations:** 1 Department of Diagnostic and Interventional Radiology, Charité-Universitätsmedizin Berlin, corporate member of Freie Universität Berlin, Humboldt-Universität Zu Berlin, and Berlin Institute of Health, Augustenburger Platz 1, 13353 Berlin, Germany; 2 Division of Gastroenterology and Hepatology, Department of Medicine, Charité-Universitätsmedizin Berlin, corporate member of Freie Universität Berlin, Humboldt-Universität Zu Berlin, and Berlin Institute of Health, Augustenburger Platz 1, 13353 Berlin, Germany; 3 Department of Diagnostic and Interventional Radiology, Universitätsklinikum Leipzig, Leipzig, Germany

## Correction to: Abdominal Radiology (2021) 46:979–991 10.1007/s00261-020-02731-z

The article “Gadoxetic acid-enhanced MRI in primary sclerosing cholangitis: added value in assessing liver function and monitoring disease progression”, written by Aboelyazid Elkilany, Dominik Geisel, Tobias Müller, Andreas Fischer, Timm Denecke, was originally published electronically on the publisher’s internet portal on 12 September 2020 without open access. With the author(s)’ decision to opt for Open Choice the copyright of the article changed on 25 May 2021 to © The Author(s) 2020 and the article is forthwith distributed under a Creative Commons Attribution 4.0 International License, which permits use, sharing, adaptation, distribution and reproduction in any medium or format, as long as you give appropriate credit to the original author(s) and the source, provide a link to the Creative Commons licence, and indicate if changes were made. The images or other third party material in this article are included in the article’s Creative Commons licence, unless indicated otherwise in a credit line to the material. If material is not included in the article’s Creative Commons licence and your intended use is not permitted by statutory regulation or exceeds the permitted use, you will need to obtain permission directly from the copyright holder. To view a copy of this licence, visit http://creativecommons.org/licenses/by/4.0.

The original article has been corrected.

