# Supplementary material for: Gadoxetic acid-enhanced MRI in primary sclerosing cholangitis: added value in assessing liver function and monitoring disease progression
Source: Abdom Radiol (NY). 2020 Sep 12;46(3):979–91. doi: 10.1007/s00261-020-02731-z (PMC8257540; doi:10.1007/s00261-020-02731-z)
Supplement: Supplementary file 1 — Supplementary material 1 (DOCX 48 kb) [file 261_2020_2731_MOESM1_ESM.docx]

| **A** | | | | | | | | | | | |
| --- | --- | --- | --- | --- | --- | --- | --- | --- | --- | --- | --- |
|  | | **Unenhanced T2WIs** | | | | | **T2-weighted MRCP** | | **T1WIs (VIBE FS)** | | |
|  |  | HASTE | FS TSE | | | | THASTE FS Thick slab | SPACE | before contrast | HBP | HBP |
| **Slice thickness (mm)** | | 6 | 6 | | | | 50 | 1 | 3 | 3 | 3 |
| **Matrix** | | 256X256 | 512X264 | | | | 384X269 | 384x353 | 276x340 | 276x340 | 276x340 |
| **TR (ms)** | | 1400 - 1600 | 5737- 6396 | | | | 4500 - 6000 | 5824 | 4,58 | 4,58 | 4,58 |
| **TE (ms)** | | 90 - 93 | 100 -102 | | | | 735 - 900 | 698 | 2,25 | 2,25 | 2,25 |
| **Imaging plane** | | axial | Axial | | | | coronal | coronal | axial | Axial | Coronal |
| **Flip angle (°)** | | 150 | 150 | | | | 180 | 140 | 9 | 9 | 9 |
| **B** | | | | | | | | | | | |
|  | | **Unenhanced T2WIs** | | | | | **T2-weighted MRCP** | | **T1WIs (VIBE)** | | |
|  |  | HASTE | FS TSE | | | | THASTE FS Thick slab | SPACE | before contrast | HBP | HBP |
| **Slice thickness (mm)** | | 6 | 6 | | | | 50 | 1 | 3 | 3 | 3 |
| **Matrix** | | 256X256 | 256X256 | | | | 384X269 | 384x353 | 276x340 | 276x340 | 276x340 |
| **TR (ms)** | | 1400 - 1600 | 3600 – 5400 | | | | 4500 - 6000 | 5824 | 4,25 | 4,25 | 4,25 |
| **TE (ms)** | | 90 - 93 | 100 -102 | | | | 735 - 900 | 698 | 2,07 | 2,07 | 2,07 |
| **Imaging plane** | | axial | Axial | | | | coronal | coronal | axial | Axial | Coronal |
| **Flip angle (°)** | | 150 | 150 | | | | 180 | 140 | 9 | 9 | 9 |
| **C** | | | | | | | | | | | |
|  | **Unenhanced T2WIs** | | | | **T2-weighted MRCP** | | | | **T1WIs (LAVA)** | | |
|  | SSFSE | | FRFSE FS | | 2D SSFSE | | | 3D FRFSE | before contrast | HBP | HBP |
| **Slice thickness (mm)** | 6 | | 6 | | 40 | | | 2,8 | 5 | 5 | 5 |
| **Matrix** | 320X256 | | 320X224 | | 320X265 | | | 256X224 | 320X192 | 320X192 | 320X192 |
| **TR (ms)** | 2625 | | 3000 | | 8000 | | | 5455 | 5,5 | 5,5 | 5,5 |
| **TE (ms)** | 90 | | 72 | | 1198 | | | 549 | 2,7 | 2,7 | 2,7 |
| **Imaging plane** | axial | | Axial | | coronal | | | coronal | axial | Axial | Coronal |
| **Flip angle (°)** | 90 - 150 | | 90 - 150 | | 90 - 180 | | | 90 - 180 | 9 | 9 | 9 |
| **D** | | | | | | | | | | | |
|  | **Unenhanced T2WIs** | | | | | **T2-weighted MRCP** | | | **T1WIs (WATS)** | | |
|  | UTSE | | | FS TSE | | SSh RAD Thick Slab | | 3DHR SENSE | before contrast | HBP | HBP |
| **Slice thickness (mm)** | 7 | | | 7 | | 40 | | 1 | 5 | 5 | 5 |
| **Matrix** | 256X187 | | | 400X299 | | 256X205 | | 226X204 | 224X157 | 224X157 | 224X157 |
| **TR (ms)** | 2100 | | | 1600 | | 8000 | | 1151 | 110 | 110 | 110 |
| **TE (ms)** | 90 | | | 100 | | 800 | | 650 | 6.9 | 6.9 | 6,9 |
| **Imaging plane** | axial | | | Axial | | coronal | | coronal | axial | Axial | Coronal |
| **Flip angle (°)** | 150 | | | 150 | | 90 | | 90 | 10 | 10 | 10 |

**Supplementary table 1.** Sequences parameters used for MRI examinations on the different MRI machines. (A) Sequence parameters for 1.5T Siemens Magnetom Avanto and 1.5T Siemens Magnetom Aera; (B) sequence parameters for 3.0T Siemens Magnetom Skyra and 3.0T Biograph mMR (Siemens Healthcare, Erlangen, Germany). (C) Sequence parameters for 1.5T GE Signa Excite (GE Medical Systems, Milwaukee, WI, USA) and (D) sequence parameters for 1.5T Intera (Philips, Best, The Netherlands).

**FS:** fat suppression. **FRFSE:** fast-recovery fast spin-echo. **[HASTE](https://radiopaedia.org/articles/missing?article%5Btitle%5D=haste&lang=gb):** half-Fourier acquisition single-shot turbo spin-echo. **LAVA**: liver acquisition with volume acquisition. **SSFSE:** single-shot fast spin echo. **SPACE:** sampling perfection with application-optimized contrasts using different flip angle evolution (Siemens healthcare). **SSh MRCP Rad:** single-shot MRCP radial sequence. [**TSE**](https://radiopaedia.org/articles/fast-spin-echo?lang=gb)**:** turbo spin echo. **UTSE:** ultra-turbo spin echo. **VIBE:** volumetric interpolated breath-hold sequence. **WATS:** water selective. **3DHR:** three-dimensional high resolution.

|  | **0** | **1** | **2** | **3** |
| --- | --- | --- | --- | --- |
| **Degree of bile duct dilatation.** | No  < 3 mm | Minimal  3 – 4 mm | Mild  4 – 5 mm | Marked  > 5 mm |
| **Bile duct caliber irregularity. *** | No | Yes | - | - |
| **Significant bile duct stenosis.** | No | Yes | - | - |
| **HBP gadoxetic acid excretion into dilated segmental bile ducts.** | No | Yes | - | - |
| **Parenchymal T2 hyperintensity.** | No | Partial / Subsegmental | Total / Segmental | - |
| **Parenchymal contrast enhancement in HBP.** | No | Partial / Subsegmental | Total / Segmental | - |
| **Segmental atrophy.** | No | Partial /Subsegmental | Total / Segmental | - |

**Supplementary table 2.** Presence and severity of different qualitative MRI imaging features at segmental level. HBP: hepatobiliary phase.

*Bile duct caliber irregularity: beading (multiple segmental caliber irregularities in the form of strictures alternating with dilatations) and pruning (peripheral bile duct attenuations).

|  | **N** | **Signal intensity before contrast** | | **Signal intensity in HBP** | | **Relative enhancement** | |
| --- | --- | --- | --- | --- | --- | --- | --- |
|  |  | **Mean±SE** | **95% CI** | **Mean±SE** | **95% CI** | **Mean±SE** | **95% CI** |
| **Segment 1** | 227 | 218.04±6.94 | 205.65 – 235.43 | 361.57±13.20 | 335.69 – 392.13 | .64±.02 | 0.59 - 0.67 |
| **Segment 2** | 214 | 203.37±6.87 | 189.93 – 217.97 | 312.15±12.39 | 287.56– 338.19 | .51±.02 | 0.47 - 0.54 |
| **Segment 3** | 205 | 202.48±7.42 | 188.30 – 217.65 | 308.64±12.52 | 284.78 – 334.26 | .50±.02 | 0.47 - 0.54 |
| **Segment 4** | 227 | 208.06±7.09 | 195.36 – 225.76 | 328.03±12.16 | 302.02 – 353.68 | .56±.02 | 0.51 - 0.58 |
| **Segment 5** | 227 | 221.71±7.77 | 206.75 – 240.04 | 369.42±13.74 | 339.73 – 398.25 | .66±.02 | 0.61 - 0.69 |
| **Segment 6** | 227 | 220.62±6.83 | 208.14 – 237.30 | 366.94±12.48 | 341.07 – 393.59 | .65±.02 | 0.60 - 0.68 |
| **Segment 7** | 227 | 230.30±7.33 | 216.55 – 247.92 | 362.12±12.64 | 334.50 – 387.81 | .56±.02 | 0.51 - 0.58 |
| **Segment 8** | 227 | 220.95±7.94 | 206.81 – 240.95 | 343.67±13.77 | 316.19 – 374.97 | .54±.02 | 0.49 - 0.57 |
| **Right lobe** | 227 | 222.57±7.34 | 209.09 – 240.55 | 358.96±12.94 | 332.0 – 386.86 | .59±.02 | 0.55 - 0.61 |
| **Left lobe** | 227 | 206.37±6.72 | 194.32 – 223.21 | 326.10±12.00 | 301.24 – 352.64 | .55±.02 | 0.50 - 0.57 |
| **Whole liver** | 227 | 214.32±6.70 | 201.44 – 231.46 | 342.07±12.40 | 315.95 – 368.74 | .57±.02 | 0.52 - 0.59 |

**Supplementary table 3.** Summary of descriptive results for quantitative signal intensity measurements of liver parenchyma in patients with PSC.

HBP, hepatobiliary phase; RE, relative enhancement; SE, standard error; CI, confidence interval.

|  | **Linear mixed model** | | | | | | | | | | | |
| --- | --- | --- | --- | --- | --- | --- | --- | --- | --- | --- | --- | --- |
|  | *n* | Sig. | **Sig. of pairwise comparison** | | | | | | | | | |
|  |  |  | MRI 1 to 2 | MRI 1 to 3 | MRI 1 to 4 | MRI 1 to 5 | MRI 2 to 3 | MRI 2 to 4 | MRI 2 to 5 | MRI 3 to 4 | MRI 3 to 5 | MRI 4 to 5 |
| **RE** | 183 | < .001 | **0.03^*^** | 1.000 | 1.000 | 1.000 | 1.000 | 1.000 | 1.000 | 1.000 | 1.000 | 1.000 |
| **RE in excretory group** | 133 | < .001 | **0.001^*^** | 1.000 | 1.000 | 1.000 | 1.000 | 1.000 | 1.000 | 1.000 | 1.000 | 1.000 |
| **Amsterdam-Oxford model** | 73 | < .001 | 1.000 | 1.000 | 1.000 | 0.213 | 1.000 | 1.000 | 0.371 | 1.000 | 0.620 | 0.066 |
| **MELD score** | 164 | < .001 | 1.000 | 1.000 | 1.000 | 1.000 | 1.000 | 1.000 | 1.000 | 1.000 | 1.000 | 1.000 |
| **Mayo risk score** | 77 | < .001 | 1.000 | 1.000 | 1.000 | 1.000 | 1.000 | 1.000 | 1.000 | 1.000 | 1.000 | 1.000 |
| **Bilirubin** | 172 | 0.02 | 1.000 | 1.000 | 1.000 | 1.000 | 1.000 | 1.000 | 1.000 | 1.000 | 1.000 | 1.000 |
| **INR** | 162 | < .001 | 1.000 | 1.000 | 1.000 | 1.000 | 1.000 | 1.000 | 1.000 | 1.000 | 1.000 | 1.000 |
| **Albumin** | 79 | 0.006 | 1.000 | 1.000 | 1.000 | **0.03^*^** | 1.000 | 1.000 | **0.02^*^** | 0.760 | 0.457 | 0.430 |
| **ALP** | 167 | < .001 | 1.000 | 1.000 | 1.000 | 1.000 | 1.000 | 1.000 | 1.000 | 1.000 | 1.000 | 1.000 |
| **GGT** | 169 | < .001 | 1.000 | 1.000 | 1.000 | 1.000 | 1.000 | 1.000 | 1.000 | 0.630 | 0.448 | 0.970 |
| **AST** | 167 | < .001 | 1.000 | 1.000 | 1.000 | 1.000 | 1.000 | 1.000 | 1.000 | 1.000 | 1.000 | 1.000 |
| **ALT** | 169 | < .001 | 1.000 | 1.000 | 1.000 | 1.000 | 1.000 | 1.000 | 1.000 | 1.000 | 1.000 | 1.000 |

**Supplementary Table 4.** Time course of relative enhancement of the liver in serial gadoxetic acid-enhanced MRI examinations in relation to clinical scores and laboratory values at the corresponding timepoints using linear mixed model with timepoint of MRI examination as fixed variable. P-value < 0.05 was considered statistically significant and is marked with *.

RE, relative enhancement; MELD, Model for End-Stage Liver Disease; AST, aspartate aminotransferase; ALT, alanine aminotransferase; ALP, alkaline phosphatase; INR, international normalized ratio; GGT, Gamma-glutamyl transferase.

|  | **MRI 1 (n=59)** | | **MRI 2 (n=59)** | | **MRI 3 (n=35)** | | **MRI 4 (n=21)** | | **MRI 5 (n=9)** | |
| --- | --- | --- | --- | --- | --- | --- | --- | --- | --- | --- |
|  | **n** | **Mean±SE** | **n** | **Mean±SE** | **n** | **Mean±SE** | **n** | **Mean±SE** | **n** | **Mean±SE** |
| Time to previous MRI in days (range) | - | – | - | 631.62±519.2  (45-2664) | - | 540.24±331.07  (87-1514) | - | 409.13±214.28  (76-835) | - | 406.31±183.15  (136-793) |
| Amount of contrast (ml) | - | 7.87±1.24 | - | 7.83±1.17 | - | 7.74±1.26 | - | 7.73±1.19 | - | 7.2±1.23 |
| Age in years (range) | - | 41.3±12.08  (20.8-61.9) | - | 43.17±12.2  (21.7-64.3) | - | 46.38±11.63  (24.05-65.8) | - | 51.2±11.2  (26.4-66.9) | - | 51.9±9.6  (29.5-63.2) |
| Gender (m/f) | 43/16 | - | 43 /16 | - | 26/9 | - | 13/8 | - | 4/5 | - |
| Liver cirrhosis | 23 | (39.00 %) | 25 | (42.37%) | 21 | (60.00%) | 13 | (61.9%) | 8 | (88.9%) |
| Contrast excretion into CBD during HBP | 45 | (76.3%) | 43 | (72.9%) | 24 | (68.6%) | 14 | (66.7%) | 7 | (77.8%) |
| RE in HBP (whole liver) | 59 | 0.64±0.31 | 59 | 0.56±0.24 | 35 | 0.57±0.24 | 21 | 0.59±0.26 | 9 | 0.57±0.39 |
| RE in HBP (seg. 1) | 59 | 0.69±0.33 | 59 | 0.56±0.23 | 35 | 0.61±0.29 | 21 | 0.63±0.29 | 9 | 0.56±0.31 |
| RE in HBP (seg. 2) | 59 | 0.58±0.27 | 59 | 0.47±0.27 | 35 | 0.48±0.27 | 21 | 0.52±0.27 | 9 | 0.45±0.33 |
| RE in HBP (seg. 3) | 59 | 0.58±0.27 | 59 | 0.48±0.24 | 35 | 0.46±0.25 | 21 | 0.49±0.27 | 9 | 0.47±0.37 |
| RE in HBP (seg. 4) | 59 | 0.58±0.28 | 59 | 0.51±0.25 | 35 | 0.59±0.29 | 21 | 0.57±0.24 | 9 | 0.62±0.31 |
| RE in HBP (seg. 5) | 59 | 0.67±0.27 | 59 | 0.64±0.29 | 35 | 0.67±0.24 | 21 | 0.59±0.37 | 9 | 0.63±0.33 |
| RE in HBP (seg. 6) | 59 | 0.67±0.27 | 59 | 0.61±0.24 | 35 | 0.65±0.27 | 21 | 0.62±0.33 | 9 | 0.59±0.31 |
| RE in HBP (seg. 7) | 59 | 0.63±0.29 | 59 | 0.48±0.25 | 35 | 0.58±0.28 | 21 | 0.59±0.31 | 9 | 0.53±0.36 |
| RE in HBP (seg. 8) | 59 | 0.59±0.29 | 59 | 0.50±0.27 | 35 | 0.56±0.25 | 21 | 0.57±0.27 | 9 | 0.54±0.29 |
| Mayo risk score | 29 | 0.33±1.31 | 26 | 0.58±1.29 | 8 | 0.72±1.21 | 10 | 0.22±1.39 | 4 | 1.27±0.97 |
| MELD score | 55 | 8 | 55 | 8 | 27 | 9 | 19 | 9 | 8 | 9 |
| Amsterdam-Oxford model | 26 | 1.96±0.66 | 25 | 2.07±0.67 | 7 | 2.07±0.66 | 11 | 1.94±0.84 | 4 | 2.45±0.44 |
| 5-year transplant-free estimated survival | 26 | 85.67±10.16 | 25 | 84.46±9.82 | 7 | 84.85±10.32 | 11 | 83.91±14.56 | 4 | 80.48±7.16 |
| 10-year transplant-free estimated survival | 26 | 68.93±17.66 | 25 | 66.45±17.71 | 7 | 67.37±18.47 | 11 | 67.12±24.06 | 4 | 57.84±12.73 |
| 15-year transplant-free estimated survival | 26 | 55.81±20.69 | 25 | 52.63±20.13 | 7 | 53.87±21.36 | 11 | 57.09±28.17 | 4 | 41.13±14.41 |
| Bilirubin (mg/dl) | 58 | 1.89±2.78 | 58 | 2.02±3.49 | 27 | 2.00±1.84 | 21 | 1.81±2.08 | 8 | 2.13±2.30 |
| AST (U/L) | 56 | 68.73±52.12 | 56 | 70.23±46.68 | 28 | 70.90±42.08 | 19 | 49.32±27.88 | 8 | 59.1±37.90 |
| ALT (U/L) | 56 | 92.7±88.18 | 56 | 86.37±88.28 | 28 | 89.64±85.45 | 21 | 62±33.34 | 8 | 55.12±28.27 |
| GGT (U/L) | 56 | 268.9±242.91 | 56 | 223.4±231.29 | 28 | 272.53±260.38 | 21 | 185.91±147.43 | 8 | 148.3±131.26 |
| ALP (U/L) | 56 | 279.33±176.19 | 56 | 262.6±179.43 | 28 | 282.27±194.71 | 19 | 263.11±277.71 | 8 | 239.2±131.74 |
| Albumin (gm/L) | 29 | 4.03±0.63 | 26 | 4.02±0.54 | 11 | 4.08±0.48 | 9 | 4.03±0.66 | 4 | 3.23±0.33 |
| Platelets (x109/L) | 57 | 258.7±100.3 | 61 | 229.90±101.27 | 28 | 217.46±119.72 | 20 | 239.44±94.5 | 8 | 202.5±48.73 |
| INR | 54 | 1.04±0.14 | 53 | 1.05±0.18 | 29 | 1.05±0.12 | 18 | 1.08±0.20 | 8 | 1.05±0.12 |
| Creatinine (mg/dl) | 57 | 0.77±0.15 | 54 | 0.78±0.15 | 31 | 0.80±0.15 | 20 | 0.77±0.16 | 9 | 0.75±0.14 |
| eGFR (ml/min) | 34 | 87.05±8.13 | 46 | 88.64±4.9 | 29 | 106,82±4.8 | 17 | 87.5±6.72 | 9 | 87.22±3.82 |
| CRP (mg/l) | 30 | 10.96±21.37 | 46 | 13.8±20.4 | 22 | 12.27±20.33 | 13 | 7.69±9.85 | 6 | 7.2±8.10 |
| Leucocytes(x109/L) | 57 | 7.45±3.16 | 576 | 6.46±2.5 | 27 | 5.88±1.76 | 19 | 6.41±1.73 | 8 | 5.64±1.17 |

**Supplementary Table 5.** Results of descriptive analysis of patients with at least two gadoxetic acid-enhanced MRI examinations.

ALT, alanine aminotransferase; AST, aspartate aminotransferase; ALP, alkaline phosphatase; CRP, C-reactive protein; eGFR, estimated glomerular filtration rate; GGT, gamma-glutamyl transferase; HBP, hepatobiliary phase; INR, international normalized ratio; MELD, Model for End-Stage Liver Disease.
